# Advancing quality and safety of perinatal services in India: opportunities for effective midwifery integration

**DOI:** 10.1093/heapol/czac032

**Published:** 2022-04-16

**Authors:** Saraswathi Vedam, Reena Titoria, Paulomi Niles, Kathrin Stoll, Vishwajeet Kumar, Dinesh Baswal, Kaveri Mayra, Inderjeet Kaur, Pandora Hardtman

**Affiliations:** Department of Family Practice, University of British Columbia, 304-5950 University Blvd, Vancouver, BC V6T 1Z3, Canada; Population Health Observatory, Fraser Health Authority, Suite 400, Central City Tower 13450 – 102nd Avenue, Surrey, BC V3T 0H1, Canada; Rory Meyers College of Nursing, New York University, 433 1st Avenue, New York, NY 10010, USA; Department of Family Practice, University of British Columbia, 304-5950 University Blvd, Vancouver, BC V6T 1Z3, Canada; Community Empowerment Lab, 26/11 Wazir Hasan Road, Gokhale Marg, Lucknow, UP 226001, India; MAMTA Health Institute for Mother and Child, B-5, Greater Kailash Enclave-II, New Delhi 110048, India; Global Health Research Institute, Faculty of Social Sciences, University of Southampton, University Road, Southampton SO17 1BJ, UK; Fernandez Foundation, Fernandez Hospital, 4-1-120, Bogulkunta, Hyderabad 500001, India; Johns Hopkins Program for International Education in Gynecology and Obstetrics, John Hopkins University, 1615 Thames Street, Baltimore, MD 21231, USA

**Keywords:** Health services, integration, national health service, policy implementation, pregnancy, nurse practitioners, mothers, maternity services, community health

## Abstract

India has made significant progress in improving maternal and child health. However, there are persistent disparities in maternal and child morbidity and mortality in many communities. Mistreatment of women in childbirth and gender-based violence are common and reduce women’s sense of safety. Recently, the Government of India committed to establishing a specialized midwifery cadre: Nurse Practitioners in Midwifery (NPMs). Integration of NPMs into the current health system has the potential to increase respectful maternity care, reduce unnecessary interventions, and improve resource allocation, ultimately improving maternal–newborn outcomes. To synthesize the evidence on effective midwifery integration, we conducted a desk review of peer-reviewed articles, reports and regulatory documents describing models of practice, organization of health services and lessons learned from other countries. We also interviewed key informants in India who described the current state of the healthcare system, opportunities, and anticipated challenges to establishing a new cadre of midwives. Using an intersectional feminist theoretical framework, we triangulated the findings from the desk review with interview data to identify levers for change and recommendations. Findings from the desk review highlight that benefits of midwifery on outcomes and experience link to models of midwifery care, and limited scope of practice and prohibitive practice settings are threats to successful integration. Interviews with key informants affirm the importance of meeting global standards for practice, education, inter-professional collaboration and midwifery leadership. Key informants noted that the expansion of respectful maternity care and improved outcomes will depend on the scope and model of practice for the cadre. Domains needing attention include building professional identity; creating a robust, sustainable education system; addressing existing inter-professional issues and strengthening referral and quality monitoring systems. Public and professional education on midwifery roles and scope of practice, improved regulatory conditions and enabling practice environments will be key to successful integration of midwives in India.

Key messages

- There are a number of opportunities and threats to integration of midwives in India, including regulatory and educational structures; role, scope and models of practice; inter-professional and public acceptance and enabling practice environments.
- Gender issues and marginalization impact the delivery and organization of health care in India—for both maternity service users and health professionals—and could destabilize the new midwifery cadre.
- Expansion of training in human rights, respectful maternity care and inter-professional communication for midwives, educators and other health professionals who work alongside midwives will be essential to effective integration.
- A set of recommendations are presented to ensure that the well-being and sustainability of a new midwifery workforce are secured, along with considerations for equity in roles, compensation and leadership.

## Introduction

**Figure 1.**
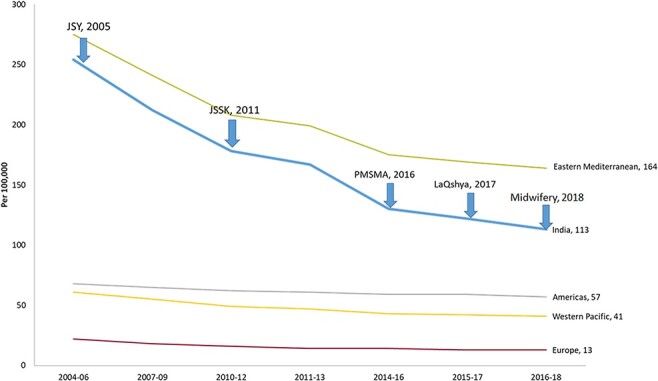
Temporal trend of MMR across different regions and introduction of flagship programmes in India (2004–2018)

Every year more than 27 million babies are born in India, comprising one-fifth of all births globally (Mavalankar *et al.*, 2008). Over the last 20 years, the country has made impressive progress in addressing high rates of mortality among mothers and newborns. Between 1990 and 2018, the maternal mortality ratio in India decreased from 556 to 113 deaths per 100 000 live births (Khetrapal, 2018; Office of the Registrar General India, 2020). The groundbreaking progress observed over the years (see Figure 1) was possible due to strong political commitment and implementation of multi-pronged initiatives under the National Health Mission (Bhatia *et al.*, 2021) and the Department of Women and Child Development. These include two formal community health worker programmes, Accredited Social Health Activists (ASHA) and Anganwadi workers; the safe motherhood intervention, Janani Suraksha Yojana (JSY), which promotes institutional delivery by conditional cash transfer to service users; and Janani Shishu Suraksha Karyakram (JSSK) that provides free entitlements for pregnant women and sick newborns. The Pradhan Mantri Surakshit Matritva Abhiyan focused on positive engagement between public and private healthcare providers, ensuring quality antenatal care and high-risk pregnancy detection in pregnant women. Finally, labour room quality improvement initiatives, LaQshya and more recently Surakshit Matritva Aashwasan (SUMAN), go beyond entitlements and focus on assuring the provision of respectful maternal healthcare services.

MMR, Source: Sample Registration System report by Registrar General of India; Global Health Observatory Data Repository; Maternal mortality estimates by WHO region.

Despite these strategies, India’s very large population and annual birth cohort still contribute more to global infant and maternal morbidity and mortality than other countries (Dandona *et al.*, 2020; Kassebaum *et al.*, 2016). Mortality rates remain high in rural areas, among women from scheduled castes, those living in urban slums and women with low socio-economic status and low health literacy (O’Neil *et al.*, 2017). In addition, there is a significant within-country variation of maternal morbidity and mortality (maternal mortality ratio, MMR). Of the 28 states and 8 Union Territories, only 5 states meet the 2015 Sustainable development goal (SDG) of an MMR of less than 70 per 100 000 live births (Office of the Registrar General India, 2020). Although institutional deliveries have doubled from 38.7% to 78.9%, this increase has not resulted in the equivalent commensurate reduction of morbidity and mortality [International Institute for Population Sciences (IIPS) and ICF, 2017]. A critical cause is the lack of improvement in the quality of care provided in public health facilities (Iyengar *et al.*, 2014). For example, even where institutional birth is available, rising rates of obstetric interventions, including induction and caesarean section, without evidence-based indications, contribute to population-wide short- and long-term morbidities that previously were uncommon in India (Singh *et al.*, 2018).

Some changes in practice, such as over-medicalization of care, can be linked to the lack of capacity to manage increased volume of cases in institutions (Miller *et al.*, 2016). India is affected by a severe shortage of skilled birth attendants (O’Neil *et al.*, 2017). In most states, a relatively small cohort of obstetricians oversees primary maternity services. Auxiliary nurses (ANMs) provide immunization services and antenatal care, and some staff nurses (GNMs) attend births; but, although called nurse-midwives, their specific education on perinatal care is limited. Poor quality care due to the lack of carer knowledge, skills and resources or unnecessary interventions directly contributes to preventable maternal and perinatal deaths (Chou *et al.*, 2019) and also to adverse clinical and psychological outcomes for the mother, baby and family (Finlayson and Downe, 2013). An overburdened workforce correlates with a decrease in supportive, empathic behaviours and an increase in negative interactions between providers and patients (Bodenheimer and Sinsky, 2014; Mayra *et al.*, 2021a). When service users encounter delays in care, disrespect and/or abuse they are less likely to seek care or adhere to recommendations, even when those are evidence based or potentially life-saving (Bohren *et al.*, 2014). Mistreatment during pregnancy and childbirth has been recognized as a form of gender-based violence, which is rooted in structural gender inequality and involves systematic devaluation of the health, safety and rights of women (Jewkes and Penn-Kekana, 2015).

In addition, given that the majority of childbearing women live in rural areas, improvements to coordination of care and the referral structure are essential. A recent comprehensive landscape analysis of maternal health in India identified opportunities for increasing supply of healthcare providers through the expansion of roles for midwives; improving community demand for services through health empowerment and health system accountability for patient experience and supporting advocacy and evidence generation by expanding partnership networks among community health workers, rural health centres and tertiary care facilities (O’Neil *et al.*, 2017).

### What Women Want

The White Ribbon Alliance, together with partner organizations, distributed a global survey asking childbearing women a single question: ‘What is your top request for your maternal and reproductive healthcare?’ Between 2017 and 2019, 1.2 million women from 114 countries participated in the ‘What Women Want’ (WWW) study. Among the most common requests were respectful and dignified care (103 584 responses); increased supply and competence of midwives and nurses and more fully functional health facilities, closer to women’s homes (White Ribbon Alliance, 2021). In India, 335 000 respondents to the survey generated over 350 000 requests for health system improvement; 20% asked for better access to health services, supplies and information, 18% requested care that is characterized by equity, respect and dignity and 13% requested better availability of health professionals, including midwives (White Ribbon Alliance, 2020).

### Why midwifery matters to India

Three different Lancet Series on Midwifery (2014), Maternal Health (2016) and Intervention to Reduce Unnecessary Caesareans (2018) concluded that ‘national investment in midwives and in their work environment, education, regulation, and management … is crucial to the achievement of national and international goals and targets in reproductive, maternal, newborn, and child health’ (Shrivastava and Sivakami, 2020). In December 2018, the Government of India (GoI) committed to the establishment of a midwifery cadre called Nurse Practitioners in Midwifery (NPMs) that met standards set by the International Confederation of Midwives. Their intention is to provide a model of care that is associated with optimal outcomes and improvements in patient experience (Ministry of Health and Family Welfare, 2018), while continuing to offer specialist referral care by obstetricians when needed. The GoI released the ‘Guidelines on Midwifery Services in India’, which lay out plans to train and implement NPMs at-scale to provide perinatal services at midwifery-led care units in hospitals across the country (Ministry of Health and Family Welfare, 2018). Midwifery care meets the triple aims of health system improvement (Sidhu *et al.*, 2020; Bisognano *et al.*, 2014), i.e. good population outcomes, positive experiences of care reported by service users and cost savings. The hope is that integration of professional midwives across the health system may reduce the overuse of interventions, facilitate cost-effective allocation of health human resources and improve experience of care (Ministry of Health and Family Welfare, 2018).

The Lancet systematic reviews identified 56 different outcomes that could be improved solely by adding professional midwives to the healthcare team and shifting from fragmented maternal and newborn care provision to a whole-system approach with multidisciplinary teams (Betrán *et al.*, 2018; Renfrew *et al.*, 2014; Homer *et al.*, 2014; Van Lerberghe *et al.*, 2014; Ten Hoope-Bender *et al.*, 2014). When midwives coordinate care across the continuum, health systems report lower costs through the optimal use of interventions, improved patient experience and reduced duplication of resources (Darmstadt *et al.*, 2014; Kennedy *et al.*, 2020; Stenberg *et al*., 2016). The projected effect of scaling up midwifery in 78 countries observed that about 30% of maternal deaths could be averted through scaling up midwives alone, with additional maternal deaths averted on the inclusion of specialist medical care (Homer *et al.*, 2014).

To date, many of these benefits have not been realized in low- and middle-resource countries (LMICs) due to unclear guidelines on midwifery scope of practice (Sharma *et al.*, 2013), education, regulation and integration into existing health systems. Facilities that provide enabling environments for midwives to offer their expertise across the continuum of perinatal services are scarce. Case studies in Brazil, China and Chile demonstrated the tendency of health systems to rely on the routine use of medical interventions to improve maternal and newborn outcomes, believing that a focus on managing (rare) obstetric emergencies can result in a reduction in maternal and perinatal mortality (McDougall *et al.*, 2016). However, without the balancing effect of services and model offered by primary midwifery care, this strategy has resulted in rapidly growing rates of unnecessary and expensive interventions, such as caesarean sections, and inequalities in the provision of care and outcomes (Van Lerberghe *et al.*, 2014; Ten Hoope-Bender *et al.*, 2014). Also, increasing the coverage of emergency services alone does not guarantee a reduction in maternal and newborn morbidity and mortality or improvements in community trust, uptake and experience of care.

Improving access to skilled providers (primary and specialist) and quality of care are equally important; midwives can serve as the essential link in the ‘continuum of care’, from community to a functioning referral facility. Although there is now robust global literature supporting the efficacy of midwifery [Homer *et al.*, 2014; Ten Hoope-Bender *et al.*, 2014; World Health Organization (WHO), 2016a] and calling for expansion of professional midwives in LMICs, information on how to actualize this goal and country-specific guidance around implementation of optimal regulatory, educational and health systems structures are not available. In this paper, our aim is to inform quality improvement and policy initiatives that seek to establish the most effective midwifery workforce in India. We synthesize knowledge, evidence and lessons learned from other countries that have previously added midwives to the register of health professionals, as well as insights from current clinicians, public health and patient experience experts in India.

## Methods

Our overall goal was to identify current opportunities and potential barriers to integration of a midwifery workforce in India and elicit best practices from global exemplars that are applicable and relevant to public health priorities and trends in India (Mattison *et al.*, 2020). To arrive at evidence-based and actionable recommendations for policymakers, health systems leaders and educators, we utilized a modified desk review process and confirmed findings with in-country expert informants.

Desk reviews are often utilized to assess the quality of facility-level health data (WHO, 2020) or to better understand health system issues prior to undertaking field work (Agency US, Development I, Government I *et al.*, 2022). The latter approach involves a synthesis of available information and evidence on a given topic, using a three-step process: first, an environmental scan of relevant reports and articles is undertaken to provide an overview of the health system and policy environment and the key players involved. This information is then augmented with a secondary analysis of publicly available data, providing an annotated reference list that offers context for future field work (Agency US, Development I, Government I *et al.*, 2022).

The content of this desk review is based on information collected through a review of the available literature relevant to organization of maternity care and midwifery services. Primary research linking midwifery integration to outcomes; resources on midwifery health services implementation in high-, low- and middle-resource countries and patient-oriented research on quality improvement in maternity care were included. The review also included policy documents on existing arrangements for maternity service delivery and midwifery education and training in different country contexts. The desk review began with searches of academic databases focusing on peer-reviewed literature (LILACS, PsycINFO, MEDLINE and Web of Science). Keywords included midwi* AND models of education, midwi* AND Organizational model, midwi* AND Prinatal care OR Postnatal care OR Perinatal care, midwi* AND delivery of healthcare, midwi* AND multidisciplinary care team, midwi* AND Health care reform and midwi* AND continuity of patient care. We included grey literature from stakeholders’ websites and information shared by organizations working in India. Additional sources, including country-specific policy reports and regulatory guidance, were identified by midwifery leaders and networking partners at international meetings. Searches of all databases, including resources obtained from personal communications, yielded 56 relevant peer-reviewed articles, reports and regulations related to midwifery (see Supplementary Table S1).

These articles were then reviewed in full and summarized by all members of the research team. The data gathered and key issues identified from the team discussions informed the development of a guiding outline for topic-specific synthesis of the literature. Four authors synthesized the best available information for each topic in the areas of their expertise. Three authors designed visual data displays for key concepts, summary of best practices and practice models. All co-authors reviewed the synthesis, edited and provided on-the-ground relevance and contextual content.

Based on themes and gaps identified during the desk review, we consulted 10 experts engaged in health systems planning and service delivery in India. Key informant interviews were conducted to help the authors prioritize and structure findings that emerged from the desk review. Key informants (KIs) were identified from a comprehensive list of stakeholders involved in improving the quality of maternal and child health services and development of a midwifery cadre in India. The criteria for selecting the KIs were based on two factors: informant experience and involvement in the subject area and institutional and professional reputation in issues related to maternal and child health services provision. The KIs included clinicians, public health professionals, community and human rights advocates and exemplars from health system sectors involved in preparing the SUMAN operational guidelines (Ministry for Health and Family Welfare, 2020). Our multidisciplinary team developed an interview guide and included questions about the current healthcare system, relevance of the desk review findings, potential challenges and barriers to developing this new cadre and areas needing further support, investment and development. Each interview was conducted over a virtual platform and lasted between 60 and 75 min. The interviews were audio- and video-recorded and transcribed using voice recognition software. All transcripts were analysed using principles from thematic analysis by our lead qualitative researcher (Braun and Clarke, 2006). Finally, we included strategies to increase qualitative rigour such as member checking, to ensure the themes that emerged from the descriptive qualitative analysis accurately described the points raised by interviewees (Creswell and Miller, 2000).

**Figure 2.**
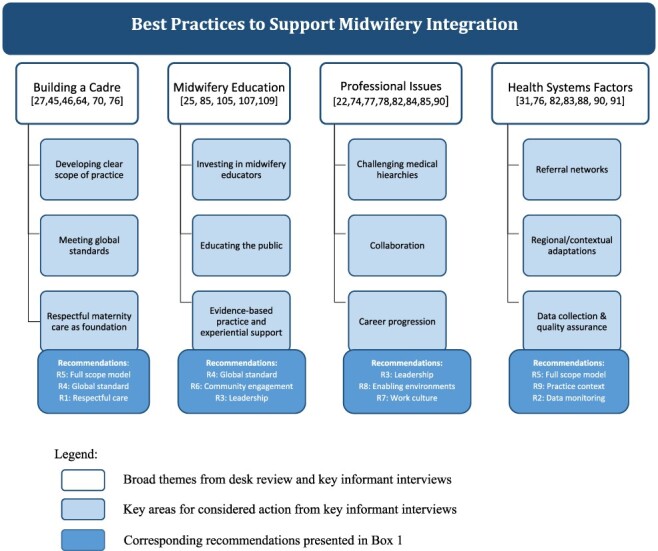
Areas for considered action in the plan for midwifery integration in India, based on desk review evidence and qualitative findings

Source: Mayra, Padmadas and Matthews (Kristensen *et al.*, 2005), licensed under CC by 4.0.SNC, State Nursing Council; ICN, International Council of Nurses.

Another confirmatory technique was the use of triangulation, i.e. ‘search for convergence among multiple and different sources of information to form themes or categories’ (McDougall *et al.*, 2016, p. 126). The senior author triangulated the data to harmonize the findings from the desk review with the qualitative interviews to identify levers for change and recommendations (see Figure 2). The outputs of this modified desk review extend beyond an annotated bibliography but serve a similar goal as traditional desk review, i.e. to supply health policymakers with relevant context and information to achieve successful integration of midwives in India.

The goal of both the desk review and confirmatory interviews was to synthesize and inform quality improvement programmes and policy initiatives that focus on implementation and integration of midwives. Hence, this project was exempt from Institutional Ethics Board review (U.S. Department of Health and Human Services, 2022). The interview guide asked participants to comment on the relevance of policy and process issues that emerged in the desk review, as well as lessons learned through local implementation of midwives. Regardless of formal ethics board clearance, appropriate safeguards were upheld: KIs were informed about the objectives of the project and their right to decline participation, and they provided informed consent prior to participating in an interview. KIs were identified by profession only, and interview data were de-identified to protect confidentiality.

### Feminist/intersectional frameworks

We explicitly use intersectional feminist theory to guide this work (Hankivsky *et al.*, 2010; Hill Collins, 2019). Any analysis of maternity care systems requires careful consideration of how gender and marginalization influence how health care is organized and delivered. Intersectionality offers a framework that considers how the multiple sources of disadvantage and discrimination impact people’s lives when confronted with the ways oppressive structures are built into the fabric of societies.

In this case, increasing access to high-quality, evidence-based midwifery services is one strategy that can address structural deficits, because the midwifery model of care has been documented as meeting the needs of those most vulnerable to poor health outcomes (Betrán *et al.*, 2018; Renfrew *et al.*, 2014; Homer *et al.*, 2014; Van Lerberghe *et al.*, 2014; Ten Hoope-Bender *et al.*, 2014).To address the intersecting factors that contribute to these outcomes beyond the clinical episode, we consider gender discrimination, economic insecurity, personal history, caste and inaccessible healthcare facilities as factors that compound risk in pregnancy and childbirth. Thus, as we present our summary findings, we centre the most marginalized service users’ priorities for quality improvement (freedom from mistreatment and equitable access) and place them in the context of a model of care that is known to enhance patient experience while achieving optimal outcomes. Then we focus on how organization of care, scope of practice, inter-professional collaboration, regulatory structures and environmental conditions for midwifery practice can affect quality, safety and efficacy.

## Results

### Desk review

#### Human rights during childbirth in India

The WHO has codified positive experiences of care as core to the well-being of childbearing women and families (WHO, 2016b). They describe standards that ensure emotional and physical support, preservation of dignity and autonomy, freedom from mistreatment and equitable access to high-quality perinatal services. The Office of the Human Rights Commissioner, with publication of its Reflection Guides (Office of the United Nations High Commissioner for Human Rights, Harvard FXB Center for Health and Human Rights, the Partnership for Maternal, Newborn and Child Health, the United Nations Population Fund and the WHO, 2016) for all levels of health workers, suggests that freedom from mistreatment and disrespect are important health outcomes in their own right. Kind, supportive and respectful perinatal care is synonymous with quality care and is linked to improved short- and long-term maternal and infant outcomes (WHO, 2016b).

Discrimination, disrespect and abuse during pregnancy and birth, on the other hand, can have long-lasting negative effects on the health and well-being of a person, family and community and compound loss of power and agency (Miller *et al.*, 2016; WHO, 2016c). New global standards call for equitable provision of care that is trauma-informed and anti-oppressive, ensures unconditional positive regard throughout healthcare interactions and prioritizes informed decision-making in health services (WHO, 2016c; The Partnership for Maternal Newborn & Child Health, 2017).

In an integrative review of 16 primary research studies about gender-based violence experienced by childbearing women in India (Shrivastava and Sivakami, 2020), authors found that obstetric violence appears to be normalized in India and is more commonly experienced by childbearing people of lower social standing. They confirmed the seven categories of mistreatment proposed by Bohren *et al* (2015) (physical abuse, sexual abuse, verbal abuse, stigma and discrimination, failure to meet professional standards of care, poor rapport between women and providers, and health system conditions and constraints) and found one additional category: harmful traditional practices and beliefs. To address these troubling issues, the authors propose a multi-level framework that incorporates rights-based approaches to care, evidence-based policies, mechanisms for redress to hold health systems accountable and theoretical and practical learning around gender equality and gender-based health inequities (Shrivastava and Sivakami, 2020). Research confirms that unsatisfying and harmful relationships between clients and providers, rooted in issues of bias and discrimination, can lead to poor health outcomes (Benkert *et al.*, 2009; Gee and Ford, 2011; McLemore *et al.*, 2018). All women and especially those who face socio-economic inequities consistently report low trust (Benkert *et al.*, 2009; Peters *et al.*, 2014; Altman *et al.*, 2019) in the healthcare system and a desire for more meaningful healthcare relationships with their maternity care provider (Halbert *et al.*, 2006; Trachtenberg *et al.*, 2005; Sheppard *et al.*, 2004).

#### Midwifery model of care and outcomes

The midwifery model of care approaches pregnancy and childbirth as physiologic processes that hold multiple forms of value and meaning (personal, physical, social, religious and cultural) for individuals, families, communities and societies (Ginsburg and Rapp, 1995; Jordan, 1992; Oakley, 1980). With the ultimate goal of providing safe outcomes for the mother and infant (Carter, 2012; Center for Health Workforce Studies, 2015; Raisler and Kennedy, 2005), midwifery care is based on development of open, trusting relationships; promotion of person-centred decision-making and encouragement of self-determination and bodily autonomy (Sword *et al.*, 2012; Kennedy *et al.*, 2003; Vedam *et al.*, 2019a). Midwifery care holds the view that the ‘relationship’ between a provider and the woman ‘and’ their family is essential. High-quality research suggests that specific elements of care—such as quality of time, trusting relationships and emotional support—are deeply valued by childbearing families (Sword, 2003; Downe *et al.*, 2016). Women report that the relationship with their provider is what they consider to be the most therapeutic aspect of their maternity care (Hunter *et al.*, 2008; Hodnett *et al.*, 2011). Such relationship-based models of care are congruent with a human rights based approach that values autonomy, informed decision-making, cultural respect and humility (Leonard, 2017; Ross, 2017; Vedam *et al.*, 2017b).

Quality indicators demonstrate that midwife-led care is associated with improvements in outcomes and experience for both low-risk and higher-risk pregnancies (Renfrew *et al.*, 2014). The 2014 Lancet Series on Midwifery identified 72 effective practices within the scope of midwifery that improve the survival, health and well-being of both healthy and at-risk women and infants. Midwifery is associated with efficient use of resources when midwives are ‘educated, trained, licensed, and regulated’ and work in collaboration as part of multidisciplinary teams (Renfrew *et al.*, 2014; McRae *et al.*, 2018). These benefits of midwifery care, established by numerous investigators, are summarized in Table 1.

**Table 1.** Midwifery Outcomes: Clinical and Affective Domains

Reference	Setting and Study design	Perinatal health outcomes
(Neal *et al.*, 2019)	USA Retrospective cohort study Low-risk parous women Inter-professional care (*n* = 12 125) vs non-inter-professional care centres (*n* = 8996)	Reduced use of selected labour and birth interventions (caesarean delivery, vacuum-assisted delivery, epidural anaesthesia, labour induction and cervical ripening) Reduced maternal duration of stay Reduced overall costs associated with Certified Nurse-Midwives (CNM)-led care relative to OB-GYN-led care
(Thornton, 2017)	USA Retrospective cohort study Vaginal births Midwives attended births (*n* = 294 604) vs physicians attended (*n* = 2 117 376)	Less epidural analgesia use (odds ratio [OR], 0.54; 95% confidence interval [CI], 0.53–0.54) Significantly fewer labour inductions (OR, 0.76; 95% CI, 0.76–0.77) Significantly fewer third- or fourth-degree lacerations (OR 0.81; 95% CI 0.78–0.84) No differences in 5-min Apgar scores, neonatal seizures, anomalous neonates or those no longer living at the time of data collection
(Attanasio and Kozhimannil, 2017)	USA Retrospective, cross-sectional analysis Association between hospital-level percentage of midwives and perinatal outcomes (*n* = 164 653)	Lower odds of giving birth by caesarean (e.g. adjusted OR [aOR], 0.70; 95% CI 0.59–0.82 at a hospital with 15–40% of births attended by midwives, compared with no midwife-attended births) Lower odds of episiotomy (e.g. aOR, 0.41; 95% CI 0.23–0.74 at a hospital with more than 40% of births attended by midwives, compared with no midwife-attended births)
(Hatem *et al.*, 2009)	Cochrane Review, including 11 trials (*n* = 12 276) Midwife-led vs other models of care for childbearing women	Fewer antenatal hospitalizations (Risk ratio [RR] 0.90; 95% CI 0.81–0.99) Fewer instrumental vaginal deliveries (RR 0.86, 95% CI 0.78–0.96) Less regional analgesia (RR 0.81, 95% CI 0.73–0.91) More spontaneous vaginal births (RR 1.04, 95% CI 1.02–1.06) Less likely to experience foetal loss before 24 weeks gestation (RR 0.79, 95% CI 0.65–0.97) More likely to breastfeed (RR 1.35, 95% CI 1.03–1.76)
(Sandall *et al.*, 2016)	Cochrane Review, including 15 trials (*n* = 17 674 women) Midwife-led continuity models vs other models of care for childbearing women	Less likely to experience preterm birth less than 37 weeks (average RR 0.76; 95% CI 0.64-0.91; *n* = 13 238; studies = 8; high quality) Less likely to experience instrumental vaginal birth (average RR 0.90; 95% CI 0.83–0.97; *n* = 17 501; studies = 13; high quality) Less likely to experience foetal loss before and after 24 weeks plus neonatal death (average RR 0.84; 95% CI 0.71–0.99; *n* = 17 561; studies = 13; high quality) Women who had midwife-led continuity models of care were more likely to experience spontaneous vaginal birth (average RR 1.05; 95% CI 1.03–1.07; *n* = 16 687; studies = 12; high quality) No differences between groups for caesarean births or intact perineum
(Johantgen *et al.*, 2012)	USA Systematic review of 21 articles describing 18 studies Comparison of labour and delivery care provided by CNMs and physicians	Higher breastfeeding rates among women cared for by CNMs compared with physician Fewer episiotomies, fewer labour inductions and fewer perineal lacerations
(Souter *et al.*, 2019)	USA Retrospective cohort study Comparing midwife (*n* = 3816) vs obstetrician (*n* = 19 284) labour and birth outcomes in low-risk hospital birth cohort	Midwifery care: lower risk of caesarean delivery among nulliparous (aRR 0.68; 95% CI 0.57–0.82) and multiparous (aRR 0.57; 95% CI 0.36–0.89) patients Lower likelihood of induction of labour (RR 0.72; 95% CI 0.64–0.81) and episiotomy (RR: 0.57; 95% CI 0.43–0.74) among nulliparous women compared with obstetrician group Lower risk of operative vaginal birth in nulliparous (aRR 0.73; 95% CI 0.57–0.93) and multiparous people (aRR 0.30; 95% CI 0.14–0.63) compared with obstetrician group
(Hodnett *et al.*, 2012)	Systematic review Effects of care in an alternative institutional birth environment (i.e. hospital birth centres usually staffed by midwives) compared with care in a conventional setting 10 trials *n* = 11 795 women	The alternative institutional setting was associated with a higher likelihood of spontaneous vaginal birth (eight trials; *n* = 11 202; RR 1.03; 95% CI 1.01–1.05); breastfeeding at 6–8 weeks (one trial, *n* = 1147; RR 1.04; 95% CI 1.02–1.06); very positive views of care (two trials, *n* = 1207; RR 1.96; 95% CI 1.78–2.15) Lower likelihood of epidural analgesia (eight trials, *n* = 10 931; RR 0.80, 95% CI 0.74–0.87); oxytocin augmentation of labour (eight trials, *n* = 11 131; RR 0.77; 95% CI 0.67–0.88); instrumental vaginal birth (eight trials, *n* = 11 202; RR 0.89; 95% CI 0.79–0.99) and episiotomy (eight trials, *n* = 11 055; RR 0.83, 95% CI 0.77–0.90)
(McRae *et al.*, 2018)	British Columbia (BC), Canada Retrospective cohort study *n* = 57 872 pregnant women, with low socio-economic position	Odds of small for gestational age birth were reduced for patients receiving antenatal midwifery vs General practice physician (GP) care (aOR 0.71; 95% CI 0.62–0.82) or OB care Odds of PTB were lower for antenatal midwifery vs GP care (aOR 0.74; 95% CI 0.63–0.86) or OB patients (aOR 0.53; 95% CI 0.45–0.62) Odds of LBW were reduced for midwifery vs GP care (aOR 0.66; 95% CI 0.53–0.82) or OB patients (aOR 0.43; 95% CI 0.34–0.54)
**Experience of care domains**		
(Sandall *et al.*, 2016)	See above	Greater overall satisfaction with care
(Hatem *et al.*, 2009)	See above	More likely to feel in control during labour and childbirth (RR 1.74; 95% CI 1.32–2.30)
(McLachlan *et al.*, 2016)	Australia randomized controlled trial (RCT) *n* = 1156 allocated to caseload midwifery, *n* = 1158 to standard care (i.e. midwifery-led care with varying levels of continuity, junior obstetric care or community-based medical care)	Women in the caseload group were more positive about their overall birth experience (aOR 1.50; 95% CI 1.22–1.84) They also felt more in control during labour, less anxious and more likely to have a positive experience of pain
(Vedam *et al.*, 2017a)	BC, Canada Cross-sectional survey Sample 1 (*n* = 1344) Sample 2 (*n* = 571) Sample 3 (*n* = 190)	Higher satisfaction with decision-making ability during pregnancy, birth, after birth and with respect to newborn care among midwifery clients compared with people with GP or OB care Higher scores on measure of agency and autonomy in decision-making using reliable and valid 7-item scale
(Vedam *et al.*, 2019a)	BC, Canada Cross-sectional survey Mixed effects analysis *n* = 2051	Midwifery clients had higher scores on measure of agency and autonomy in decision-making compared with people with GP or OB care
(Vedam *et al.*, 2019b)	USA Cross-sectional survey *n* = 2700	Lower likelihood of mistreatment among people who received prenatal midwifery care (OR 0.31; 95% CI 0.25–0.40)
(Vedam *et al.*, 2017b)	Cross-sectional survey Canada (*n* = 2271) and USA (*n* = 1613)	More respectful care experienced by service users who had midwifery vs GP or OB care. Respectful care was measured with reliable and valid 14-item scale
(Logan *et al.*, 2022)	USA Cross-sectional survey *n* = 2700	Overall significant differences in pressure and non-consent to range of obstetric interventions by type of provider; midwife-led care improved clinical and care experience Stratified by race, both white (aOR 3.02; 95% CI 1.97–4.63) and Black, Indigenous and people of colour (aOR 1.98; 95% CI 1.10–3.57) were more likely to experience non-consent during perinatal care if they had a health care provider other than a midwife during birth
(Butler *et al.*, 2015)	Ireland Mixed methods design *n* = 186	Clients who received midwife-led care had higher scores on measures of satisfaction and treatment from providers, compared with obstetrician-led antenatal clinics

An analysis of four high-resource countries that reported consistently better perinatal outcomes and lower healthcare costs identified five common factors: (1) universal access to maternity care within a continuum of care framework before pregnancy to after birth; (2) a perinatal workforce that emphasizes midwifery care and inter-professional collaboration; (3) respectful care and autonomy; (4) evidence-based guidelines on the place of birth and (5) national data collection systems (Kennedy *et al.*, 2020) (see Table 2). Moreover, the benefits of midwifery care are realized when the model of service delivery ensures adequate time to build trusting respectful relationships (Kennedy *et al.*, 2003; Rooks, 1999; Walsh and Devane, 2012) with service users. However, despite compelling evidence and the subsequent policy developments in a number of countries, organizational change to enable continuity of midwifery care has been slow and, in many LMICs like India, is non-existent (Renfrew *et al.*, 2014).

**Table 2.** High-resource country profiles of midwifery roles and scope

	Australia	Canada	Netherlands	United Kingdom
Pregnancy services provider	Through antenatal clinics with midwives and/or doctors, midwifery group practices, caseload midwifery services, aboriginal health services and birth centres depending on availability. In rural areas, GPs provide pregnancy care	Physicians attend majority (90%) of births. Midwifery became regulated in 1993 and midwives attend an average of 10% of births in 8 out of 10 provinces and one territory (2.8–22%)	Organized in two echelons: midwife-led care and obstetrician-led care. Professionals in these echelons work alongside and complementary to each other. About 89% of pregnant women start with a first antenatal visit to the community midwife. At the start of delivery, about 50% of pregnant women are under the responsibility of a midwife	Antenatal care is primarily provided by midwives in antenatal clinics in the hospital or community settings and sometimes shared with GPs. Women may choose to give birth at home in an MLU or an obstetric unit
Midwife-led models	Publicly funded programmes across the country where women receive care by midwives during prenatal and postpartum phases and can plan to give birth with midwives at home or midwives at the local hospital	Models of care differ across provinces, but in most midwives work in small teams or solo to care for women in midwife-led, community-based office practices. Midwives attend births in all available settings	Midwives can choose to work as a primary care midwife providing full scope of care for women experiencing an uncomplicated pregnancy. Alternatively, midwives can choose to work within the hospital system as a clinical midwife under the responsibility of the obstetrician	All women have a midwife and function at public health facilities (birth in midwifery-led units within hospitals, alongside units or community settings)
Midwife Education	Three-year direct-entry programme (Bachelor of Midwifery); 1-2 years graduate programme after nursing (Graduate Diploma or Masters); 4-year double degree (nursing and midwifery)	Four-year programme including 3 years of continuity care model clinical placements; 3-4 days a week of antenatal clinic and intrapartum and postpartum care	Four-year midwifery degree, at higher professional education	Three-year direct-entry programme or 18-month programme after nursing (50% of this time is spent in clinical practice); Midwives are trained to the full scope of practice at the point of registration. Additional training is required to prescribe

The third of the Lancet Series on Midwifery (Ten Hoope-Bender *et al.*, 2014) examined four LMIC countries’ (Burkina Faso, Cambodia, Indonesia and Morocco) experiences with the strengthening of health systems and deployment of midwives. They described two decades of reduction of maternal and neonatal mortality since they accelerated investment in cadres of midwives. The common sequential actions that jointly contributed to improved maternal and newborn health outcomes include (1) extension of a close-to-client network of health facilities, resulting in improved access to and uptake of facility birthing and hospital care for complications; (2) scale up of the midwifery workforce to respond to the growing demand for professional birth attendants; (3) reduction of financial barriers to accessing care (equity funds, exemptions, insurance mechanisms, government reimbursement, vouchers and conditional cash transfers) and (4) initiatives to improve quality of care. The mechanisms for incorporating midwives and challenges encountered in these countries are summarized in Table 3.

**Table 3.** LMIC experiences with deployment of midwives (Van Lerberghe *et al.*, 2014)

	Morocco	Burkina Faso	Indonesia	Cambodia
**Turning point**	Competency-based midwifery training course; training capacity was raised to nine midwifery schools. Education of midwives consists of a direct-entry 3-year training system	Professionalization of childbirth: Traditional birth attendants refocused their role on preparing women for childbirth, identifying the nearest health centre as place of birth and organizing reliable transport. Targeted one midwife per 130 women of reproductive age. Training of auxiliary midwives as an interim strategy	Village midwife programme: massive scale up of access to midwives to provide a range of primary care services. The programme initially required that a midwife should receive only 1 year of midwifery training after 9 years of schooling and 3 years of nursing training; Extended to a 3-year diploma course through midwifery academies in the 1990s	1990s: Transition from administrative-based to a population-based approach: package of activities included maternal health care, with at least two midwives per health centre. 2000s: Re-opening of direct-entry midwife training schools
**Employment**	Deploy the freshly trained midwives: minimum of two midwives per health centre with a maternity ward. Midwives work at all levels with maternity wards under the supervision of GP, in both public and private secondary- and tertiary-level hospitals. Midwives are government employees; no performance-related financial incentives to complement their modest salaries	The auxiliary midwives—originally intended as a temporary solution—oriented towards a formal midwifery training curriculum with a longer education programme. Allowing midwives to move towards a management or teaching career through an additional 3-year public health training made the profession more attractive	Employment status varied—from civil servants to short-term contract staff (local or national) to private practitioners	Each health facility has at least one midwife
**Challenge**	Roles and responsibilities remain poorly defined; Midwives have no autonomy in responding to obstetric complications	Delays in obtaining care, poor referral linkages, premature discharge of women and inadequate follow-up of unresolved health problems	Inadequate supervision and deficiencies in basic training consequent to the pace of scaling up and deployment strategy. Many midwives practising at village level, in remote postings or in private practice were put to work as sole providers	Shift from midwife to doctor among the richest quintile was associated with fast-rising caesarean section rates

##### Midwife-led units

Some countries have codified midwifery models based on midwives sharing a caseload, where women receive care from a small group of midwives who offer consistent philosophy and relational continuity (Sandall *et al.*, 2016). Midwife-led units (MLUs) in hospitals are another model for integrating the profession into existing health systems and transforming maternal health (Sandall *et al.*, 2016). MLUs are spaces headed by a midwife as the primary healthcare professional and where midwives practice to their full potential and professional autonomy, providing care to healthy pregnant women. Two types of MLUs have been established globally, alongside and free-standing (Walsh *et al.*, 2018). The free-standing MLUs (FMUs) are stand-alone birthing centres, geographically separate from their host obstetric units; if intrapartum complications develop, midwives transfer the women to specialists in hospital units. In high-resource countries, FMU care results in equivalent or better outcomes than hospital-based care in low-risk women (Stapleton *et al.*, 2013; Christensen and Overgaard, 2017; Baczek *et al.*, 2020). The AMU are nested within a hospital with immediate access to operating rooms and a Neonatal Intensive Care Unit. With the introduction of AMU in South Africa, UK, China and Nepal, there were fewer inductions, fewer caesarean sections and episiotomies, less postpartum haemorrhage, fewer admissions to special care and more spontaneous vaginal deliveries (Long *et al.*, 2016). South Africa also observed a reduction in maternal deaths.

##### Scope of practice

Challenges exist in many countries that are committed to improving their maternal health outcomes to meet and surpass the SDGs. In countries where midwifery is established, the scope of practice is clearly defined by regulatory bodies, widely communicated by midwifery associations and accepted by related professions, including nurses, physicians and administrators (Smith, 2015; McFadden *et al.*, 2020). A lack of clear, unified scope of practice results in role confusion, competition among providers, workplace tension, a lack of trust across professionals, a diminishing of professional identity and both under- and over-utilization of professionals (McFadden *et al.*, 2020; WHO, 2016d; Bar-Zeev *et al.*, 2021). Research on the characteristics of midwifery full-scope practice has these defining features: (1) firm guidelines set by self-governing regulatory bodies and professional organizations, (2) clear delineation of the autonomous primary care role including the ability to initiate consultation, collaboration and transfer to specialist care on their own recognizance and (3) flexible clinical parameters (e g. practice setting and community needs) that give depth and breadth to the scope of practice (Sharma *et al.*, 2013; Schuiling and Slager, 2000).

#### Inter-professional collaboration

Collaborative maternity care promotes respectful and active participation of each discipline involved (Macdonald *et al.*, 2015). Quality improvement literature confirms that effective inter-professional collaboration is an intentional process that can be learned and supported (Raab *et al.*, 2013). There is also growing evidence that inter-professional collaboration improves patient and provider satisfaction and health outcomes and is fundamental to ensure patient safety (Raab *et al.*, 2013; Liberati *et al.*, 2019). Inter-professional collaboration also improves access to perinatal services by addressing health human resource deficits (Peterson *et al.*, 2007). The 2014 Lancet series highlights that midwifery care has the greatest effect when provided within a health system with functional mechanisms for referral and transfer to specialist care. In midwife-led continuity models of care, the midwife is the lead care provider, who remains in an active primary care role even if she initiates referral to specialized expertise, personnel or equipment when necessary and/or outside her scope.

Scaling up midwifery services with access to referral would cost US $2200 per death averted, half as much per death averted as scaling up obstetrics (Bartlett *et al.*, 2014). To realize these full benefits, each member of the collaborative team (nurses, midwives, physicians and community health workers) must be able to function without regulatory or institutional policy restrictions to their full scope and competencies (Vedam *et al.*, 2018; Behruzi *et al.*, 2017). However, traditional hospital decision-making structures can make it challenging to accommodate an approach to care that considers multiple perspectives and types of expertise.

An organization’s leadership and culture have a critical impact on whether and how collaboration between providers is accepted (Behruzi *et al.*, 2017). Many midwives report challenges when there is little inter-professional knowledge and respect for their distinct role in achieving optimum health outcomes for mother, infant and family. Facilitators for collaboration include clarity of roles, mutual respect, shared values or vision and a willingness to collaborate (Macdonald *et al.*, 2015; Waldman *et al.*, 2012; Marshall *et al.*, 2015). Barriers to collaboration include ineffective communication, resistance to change, lack of respect, gender inequality, a lack of clearly defined roles and lack of knowledge of other health disciplines (WHO, 2016d; Macdonald *et al.*, 2015).

#### Leadership

In 2021, the WHO updated global strategic directions for strengthening midwifery (WHO, 2016a). One key area of focus was changes in governance to reduce gender disparities in leadership that exist in many LMICs. At the core of these inequalities is the lack of equal representation and decision-making power both in the labour room and at the level of policymaking. Unit leaders, department heads and government officers can all set the tone with consistent messaging around the institutional and/or health system commitments to equity within the dynamics of inter-professional decision-making structures (Marshall *et al.*, 2015). Inclusion of midwives on quality improvement teams, protocol and guideline committees and continuing professional education bodies led to prioritization of person-centred care and the optimal use of interventions. As a result, in some countries, hospital systems have codified bidirectional referral mechanisms by establishing and funding onsite availability of both Midwifery Consultants and Obstetric Consultants. All decisions related to surgeries, staffing, patient cultural safety and/or options for care require seeking input of the lead midwife before proceeding (Marshall *et al.*, 2015; Althabe *et al.*, 2004).

#### Enabling environments for a sustainable midwifery workforce

Setting up midwifery services in a way that optimizes retention and enhances job satisfaction is important to support sustainability. Midwifery is a profession characterized by high levels of occupational stress and burnout, when compared with other health and human service professionals (Kristensen *et al.*, 2005). Organizing midwifery practice in a way that supports individual practitioners has a positive effect on quality of health care. Moderate to high burnout and poor emotional health have been linked to patient safety outcomes (Hall *et al.*, 2016; Shanafelt and Noseworthy, 2017), influence patient satisfaction (Hall *et al.*, 2016) and preface midwives’ intentions to leave the profession (Stoll and Gallagher, 2019). Occupational stress and burnout are systemic issues and strongly linked to the lack of institutional support (Shanafelt and Noseworthy, 2017; Royal College of Midwives, 2016; Stoll and Butska, 2020; Cramer and Hunter, 2018).

Cramer and Hunter reviewed global evidence on the relationships between working conditions and emotional well-being of midwives (Cramer and Hunter, 2018). The authors included 44 primary research studies (22 describing quantitative data and 17 describing qualitative data) about factors associated with burnout, stress, coping and related constructs. Sidhu *et al.* performed a global scoping review of factors linked to burnout in midwifery (Sidhu *et al.*, 2020). Their review included 27 quantitative studies, and authors identified several interrelated factors that were associated with the emotional well-being of midwives. Low staffing, high workload and long hours were identified as factors contributing to burnout and loss of well-being among midwives (Sidhu *et al.*, 2020; Cramer and Hunter, 2018). Low autonomy over working patterns, low clinical autonomy and models of care that do not enable midwives to provide continuity of care are strongly associated with burnout and emotional well-being (Sidhu *et al.*, 2020; Cramer and Hunter, 2018). Working in settings that prioritize institutional needs over those of childbearing people (Sidhu *et al.*, 2020; Cramer and Hunter, 2018), difficult clinical situations, such as traumatic births, and working with clients with complex psychosocial needs are linked to reduced emotional well-being (Sidhu *et al.*, 2020; Cramer and Hunter, 2018). The relationship with colleagues plays a large role in emotional well-being. Midwives who are bullied or experience conflict with colleagues report reduced emotional well-being (Sidhu *et al.*, 2020; Cramer and Hunter, 2018).

Younger midwives with fewer years of experience are especially prone to burnout (Sidhu *et al.*, 2020; Cull *et al.*, 2020) and emotional distress. They benefit from targeted supports, like midwifery supervisors and mentors who are not involved in judging or evaluating clinical performance. Also, career development and diversification opportunities are important when planning for a sustainable midwifery profession (Sidhu *et al.*, 2020). For example, midwifery teaching, administrative or policy roles do not require on-call or night work. This may be important to individuals with chronic health problems, midwives who are themselves new parents and elder midwives. Some midwives desire opportunities for advancement in their profession. When midwives have limited opportunities for career progression or diversification of midwifery roles that align with their personal circumstances and preferences, this can intensify their emotional distress and their desire to leave the profession (Schuiling and Slager, 2000).

### Key informant interviews

#### Importance and relevance to India

The perspectives of a diverse group of KIs in India (healthcare providers, non-governmental organization leaders and government officials) confirmed and aligned with findings from our synthesis of the global literature on best practices and important considerations for midwifery integration. They posited that the midwifery model is more than just about adding a health human resource, rather it can and should lead to a paradigm shift in culture and philosophy of care. Participants placed their comments under an overarching desire to improve respectful, high-quality care through integration of a midwifery cadre. Four domains needing attention and considered action emerged: ‘how to build professional identity within a new cadre; strengthening midwifery education; inter-professional issues; and health system readiness’ (See Figure 2).

#### Building a professional cadre

##### Respectful Maternity Care

Stakeholders from various disciplinary perspectives made it clear that respectful maternity care should be the professional and ethical foundation by which an independent midwifery cadre is built. One highly skilled obstetrician-gynecologist from India suggested that respectful care is the heart of the success of the midwifery-led unit by explaining the history of this service model:

*We had to then take a 180 degree turn in our thinking – we were a group of almost 15 consultants. …. And over 9 years, we’ve made great strides … the government of xxx threw us a challenge, ‘* ***can we have compassionate, respectful care in our public hospitals?’ We took up the challenge …*** *over a two-year period, we trained these midwives that were posted in 10 districts of the state and they are creating waves, you know, they change the way things are happening, they’ve increased normal births, they’ve brought down caesarean sections, they’ve got husbands coming into the birthing room as companions, mothers are birthing in different positions. So the whole landscape has changed* (EF, OB-GYN).

Participants noted that respectful care cannot be the sole responsibility of the midwifery cadre—physicians and nurses will also benefit from understanding the client’s experiences and aspects of care interactions that may require improvement.

##### Meeting global standards for approach to midwifery practice

Our interviews with key stakeholders confirmed that global standards for midwifery practice view birth as a normal physiological process that is a critical life event for most women and mandate that midwives apply a person-centred approach to the management of normal and complex cases. There was an overall sense of optimism that adoption of the NPM model will be of benefit to Indian women and families and a caution that a lack of thoughtful attention to how midwives will be integrated into the large and complex public health system may impede the success of building this workforce (cadre) as a strategy to improve maternal health outcomes.

##### Codifying a distinct scope of practice

All KIs agreed that it will be critical that regulatory structures ensure the full scope of practice for the new cadre. One obstetric leader commented on the need for the GoI and health systems to understand the distinct professional role of midwives:

*We need to create a separate midwife cadre. You know, the governments are still looking at it, as some kind of training that you’re done with, and then they go off into labour room. They’re not understanding that it is a model and philosophy of care – that requires the continuity of care…right now, we are working backwards. We are doing the training first, and then trying to figure out, where will they go? What will they do? What is their role? How do we integrate them into health system? …* (RR).

#### Midwifery education

Given the large-scale initiative to scale up the NPM model in India, —strengthening curricula, practice education and formal mentorship to align with global standards were at the forefront of KI interviews. Creating a robust, standardized and sustainable training programme for NPMs was described as paramount to the success of building this cadre. Participants were supportive of offering both clinical skills development and foundational content on the philosophy and ethical principles that are central to the midwifery profession.

##### Consumer knowledge

KIs emphasized that it is also critical to educate service users about the midwifery model of care and the role of professional midwives, suggesting that when women begin to seek care that centres their experience and is rooted in principles of respect—then the healthcare system will need to be responsive.

*I think the endorsement of women themselves is key – if we can show talk to women about their experiences with midwives – they will tell the value of this type of care. If those voices get louder and louder, and the heads of those hospitals have to have respectful care that is assessed then we can really start to see a change* (EV).

*Education of the clients who come to you is also very important, at this moment, the things are distorted because there is an asymmetry of information between the patient and the doctor, right. Now, what has happened is that all those clients who are coming and seeing midwives are thinking ‘who are these people who are not actually applying an IV line, or giving us medication when we are admitted, they are just sitting and talking to us’*. *They don’t understand that it is because induction of labour is so rampant. So, we also have to make the consumer or the client ready for the midwives who are going to come, and educate them that they are going to give you support and all those things that are important that they are not used to expecting* (DB).

#### Inter-professional issues

KIs suggested that when midwives and physicians have the capacity to work with each other and utilize the specific knowledge and skills of each cadre, women will receive optimal care. However, collaboration also requires clear acknowledgement of the fundamental shifts in thinking about how collegial and respectful inter-professional relationships function,

*We also have to consider ‘there will be a conflict between the obstetrician and the midwife’ with the obstetricians saying ‘the case is mine, this case has been admitted under me so it is my responsibility, if anything happens – I am answerable, you are not answerable.’ So that’s a major problem. And ‘that’s why we actually opted for a collaborative care initially,’ because that builds the confidence and trust over time* (DB)

and that every player understands and appreciates their distinct roles and approaches. As one former health minister succinctly stated,

*if the medical graduate or the obstetrician tries to train the midwives, this will remove the essence of midwife and midwifery care. ‘We are building midwifery exactly because they can provide services that the obstetricians cannot’ – the current system is lacking so let’s have midwives together from the beginning so that they have their own professional community.*

##### Confronting medical hierarchy through midwifery leadership

One key aspect of any profession is how leadership is structured. Recognizing the existing medical hierarchy was a theme recounted by participants.

*We must realize that this is a hierarchical system -where the obstetricians they think they are in a higher category and because of that it is hard to have free conversations and discussion between obstetricians and midwives. So, for example, if there is a question or the midwife wants to suggest a different way – the obstetrician will say ‘no, no, no, I have explained to you, so just do what I say.’ That is not what we want* (DB).

Building midwifery leadership was seen among NPMs as a strategy to grow the profession and combat misconceptions about respective roles and expertise. One Director of Midwifery at a private hospital expressed her concern over how many obstetricians view midwives, she stated ‘until the obstetricians start believing in the midwives as colleagues, and not that we are, you know, insubordinate, I think that culture has to change’.

#### Health systems readiness

##### Strengthening the referral system

With the overall goal of improving outcomes for women and children, participants considered other primary health and triage services that midwives could provide. Many women need services for their newborns, for their own health conditions and to address the social determinants of their health. One midwifery workforce expert addressed this in their comments:

*Women need wrap around services. There needs to be simultaneous conversations actions to build up the overall care team for the woman and her child – midwifery care can serve as the centre – but let’s also look at the concurrent neonatal piece and build what is needed there as well. Can we think about build a neonatal or paediatric nurse practitioner workforce ….Ideally, we are thinking of standing up of a full perinatal workforce, that wraps around the midwife, with other roles – like community health workers – building the system and full workforce is critical. If we think the midwife can do everything – it will not give women what they need to do well when they are back home in their communities* (PH).

##### Data collection and quality assurance

Finally, all agreed that improved quality and accountability metrics may be needed.

*We need to measure respectful care and the feedback from the mothers which is different than only measuring clinical quality indicators. I’ll give you an example, I was sitting in meetings with … the government. And they were only looking at the reduction of C-sections as marker of quality care – maybe that could be one major indicator, but there are so many other things you have to look at, like what is the attention and care given by the midwife? How much time did the midwife spend with the mother? … the government needs to know what quality indicators they need to measure—we need to open our minds and eyes to see what it is that really matters and what we need to measure – not just C-sections* (RR).

## Discussion

We reviewed existing reports and literature on (a) maternal–newborn outcomes across populations following integration of midwives; (b) the impact of the model of midwifery care on access to high quality, respectful maternity care, especially among underserved and at-risk communities and (c) lessons learned regarding barriers and facilitators to integration of a dedicated, specialist cadre of midwives into a mainstream health system. Consulting with a diverse group of expert stakeholders grounded our work in the history and context of the Indian healthcare system. Our mixed methods approach allowed us to identify factors that are most important to operationalizing a robust, independent midwifery cadre that can deliver competent, compassionate care to Indian families. Research-based recommendations for assuring successful midwifery integration in India are presented in Box 1.

Box 1. Summary of recommendations: effective midwifery integration in India

**Table:** 

**MACRO—National/Federal Government**	**Operationalize human rights and respectful care for all childbearing families** **Make human rights and RMC training mandatory** for midwives, educators, and other health professionals who work alongside midwives. **Require leaders at all facilities** and midwifery-led units to engage with the OCHRC RMC Reflection Guides. **Establish incentives mechanisms for accountability**, redress, and remediation person-centred care and respectful communication at the facility level. **Build strong monitoring, data capturing and learning mechanisms** **Track maternal and newborn outcomes** related to midwifery care, including person-centred quality metrics for—respect, autonomy and mistreatment. **Map and analyse links between different models** of midwifery integration and perinatal outcomes across jurisdictions. **Collect data at point of service on patient experience** using validated person-centred measures of autonomy, respect and mistreatment. **Commit to midwifery leadership and governance** **Establish a Division of Midwifery and a Director of Midwifery,** at the highest level of the GOI—Health Affairs Unit, as well as within each state health office. This distributive leadership approach will allow for national directives to be seamlessly communicated to state departments of health and create a bidirectional flow of information, best practice, and accountability. **Build the national and state infrastructure to grow midwifery leadership within the workforce.** Include midwifery leadership at all levels of government and governance ensuring that decisions cannot be made about midwives or midwifery care, without midwives present. **Frequently engage and consult with practicing midwives** ensuring representation at all levels of organization of care.
**MESO—Regional/State Regulation**	**Reinforce midwifery education programs at global standard** **Require minimum of 18 months** of additional education in midwifery after nursing to qualify as a midwife and at least 3 years for direct entry candidates. **Establish ongoing partnerships between global and local midwifery educators** and clinician leaders to provide ongoing virtual and in person support for midwifery educators in India. **Provide mentors or supervisors for early career midwives**. Mentors/supervisors should not be in a position of power over or directly work with early career midwives. **Prioritize a full-scope relationship-based model of care for NPMs** **Build midwifery regulation that facilitates continuity of care** from preconception to early parenting. Include expanded capacity for midwives to deliver essential family planning, gynecologic, and infant care. **Establish midwives as autonomous health care providers** and support them in working to their full scope in a variety of settings. Set protocols and policies that enable midwives to participate in the care of all childbearing families. **Establish a triage network where midwives serve as primary care providers**, providing first line antepartum and intrapartum services, receiving referrals from community health workers, and initiating referrals or collaborative care plans with obstetric specialists as necessary. **Focus on community engagement and public information** **Integrate community voices** and women’s empowerment groups to define and redesign maternity care services that are respectful and responsive. **Center the needs of the most marginalized women and communities** to achieve equity. If all women and families thrive, the country can thrive. **Invest in public education and cross disciplinary information sharing** on the role, value, and benefits of midwifery care.
**MICRO—Local/Facility Environments**	**Create a positive work culture and environment for NPMs** **Ensure fair and meaningful payment structures** including parity in terms of compensation, opportunities and avenues for professional growth. **Recruit enough midwives** to manage work load and flow. **Enable role flexibility for midwives** including clinical, policy, administrative, and teaching positions **Expand organizational awareness and resources** to respond to occupational/traumatic stress. **Create enabling environments for interprofessional practice** **Educate all clinical staff on core principles of interprofessional collaboration,** including leadership models that are not hierarchical—and are respectful of different evidence streams **Promote a respectful work environment across the health professions** and mandate training on positive communication and collaboration. **Embed content on midwifery role and scope into all health professional educational programs** including medicine and nursing. **Support midwives to care for people with complex medical and social needs** **Provide additional support and incentives** to midwives who work in rural and remote settings, or who care for clients with complex care needs. **Provide additional cultural congruence training** to midwives who work with historically marginalized and at-risk populations. **Provide in-service training to community health workers and obstetric consultants** on the competencies and role of midwives in caring for special populations.

### Midwifery model and scope of practice in India

India currently lacks a cadre of professional midwives that is independent of a nursing role. The existing auxiliary nurse-midwives have not been prepared as autonomous primary care providers. Building a strong and sustainable midwifery cadre that is aligned with global standards requires role clarity such that the distinctions between ANM, NPM and nursing are explicitly articulated and delineated. Our expert informants reiterated the value of shaping the midwifery cadre in India to meet the global standards of what midwifery practice is, how they are trained and what their scope of work can entail. Using the title of midwife, without the requisite shift in how they are recognized in the healthcare system, will potentially limit their ability to improve the health and well-being of women and newborns.

Sharma’s relevant study on midwifery practice in Gujarat found that when scope of practice is not clearly defined, there will be a wide variation in that scope of practice (Sharma *et al.*, 2013). Midwives who are limited to specific intrapartum duties (changing into hospital clothes, giving an enema, shaving the perineum, placing the woman in the lithotomic position and ‘assisting’ the doctor in managing normal and complicated labour) have no independence or primary care responsibilities. The author contrasts this with ‘extended practice’ which is defined as attending to normal labour, encouraging active and respectful birth, performing repairs, addressing complications, managing third stage and postnatal care such as breastfeeding support, monitoring and managing complications and/or initiating collaborative care plans (Sharma *et al.*, 2013).

**Figure 3.**
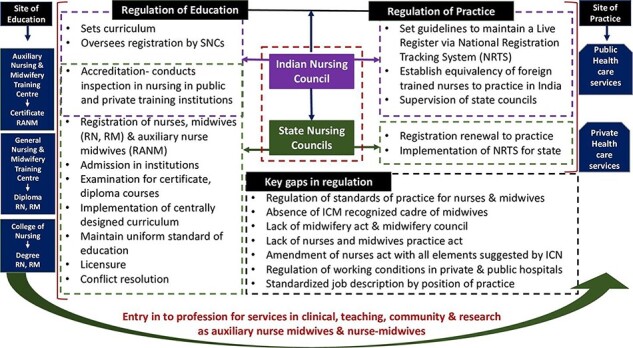
Challenges in regulation of education and practice of midwifery and nursing in India

One key issue is who will be charged with defining midwifery scope of practice. Currently, the Indian Nursing Council standardizes and monitors the quality of nursing and midwifery education. The Council has limitations in human resources and infrastructure and lacks the authority needed to address key issues that directly impact nurses and midwives (Sharma *et al.*, 2010; Mayra *et al.*, 2021b). A recent study reported on the challenges of regulating midwifery scope of education and practice across five Indian states (Figure 3), including a theory and practice mismatch in pre-service education; lack of teachers; medical domination in regulation and governance; lack of infrastructure; lack of leadership; corruption; a complete absence of regulation for private education systems and a lack of practice regulation (Mayra *et al.*, 2021b). Currently, the NPM credential is envisioned only as an advanced practice certificate issued by the Indian Nursing Council and State Nursing registration boards unlike most countries which require university preparation and thus lay the groundwork for graduate prepared midwifery educators.

The draft Nursing and Midwifery Council Bill 2020 released in November 2020 seeking feedback from the Indian citizens but has yet to be passed. This proposed bill fails to address the existing challenges and does not define the title and the scope of practice for midwives. The bill aims to replace the 74-year-old INC Act of 1947. Ideally, the Act could codify an independent midwifery cadre by establishing a regulatory Council for Midwifery and separating its role from solely implementation of the cadre in favour of an ongoing body for regulatory oversight. At the moment, the Act does not address the tenure for the president’s role, regulation of nurses and midwives practising in either the public or private sector, regulation of quality midwifery education or the role of the Council at the federal level to support and supervise the state councils. A Council for Midwifery could be the ideal body to establish uniform high standards and mechanisms for accountability by states, health officials, hospital administration, clinical leadership and midwifery providers.

### Midwifery education

Until recently the global standard for midwifery education (which includes options for building upon nursing or creating an independent cadre) was not being met in India (WHO, 2019). Both the International Confederation of Midwives (ICM) and WHO recommend following basic nursing education with 18 months of additional training in midwifery (WHO, 2005). This minimal standard will need to be universally adopted across educational programmes in India. Programmes lasting 4–5 years are most common in South-East Asia and in upper-middle-income countries. In the recently published ‘State of the World midwifery 2021’, of the 41 countries reporting a post-nursing midwifery education pathway, eight countries report a 1-year programme and three countries have a programme lasting less than 1 year (Bar-Zeev *et al.*, 2021).

Ensuring a rollout of midwifery education in India that is rooted in global standards at the central, state and district levels will be the key (McFadden *et al.*, 2020). Midwifery competencies are organized around roles, such as assessor, diagnostician, technician, counsellor, educator, communicator, collaborator and advocate (e.g. to advocate against gender-based violence) (Canadian Midwifery Regulators Council, 2020). The medicalization of pregnancy and labour in current nursing programmes often leads to a sidelining of the psychosocial and cultural value of pregnancy and birth that are important to communities. The curriculum needs to be taught by midwives who grasp and understand the midwifery philosophy of care, looking beyond a pathological perspective.

Building a midwifery cadre from a nursing background requires not only the expansion of clinical skills, but also a shift in understanding one’s professional role and responsibility. A strong independent midwifery cadre who can confidently lead midwifery care units, promote evidence-based care and provide respectful care will require learning how to own their expertise, advocate for women and reduce harm from gender-based violence. Worldwide, midwifery curricula include clinical skills development, philosophy of care, critical application of research evidence, professional responsibilities to participate in quality assurance activities and be accountable to the model and advocacy for social justice and rights. Short training programmes will not achieve these goals for role development.

Many of the state-level diploma and degree courses implemented in India for NPM registration have roughly 6 months of midwifery education. In this short duration, the students are expected to achieve the level of competence to provide pregnancy, childbirth and postnatal primary care. The educational institutions face challenges in securing adequate clinical hands-on practice for midwifery students. Given the gaps in basic education, it can be assumed that government and private sector spending includes a large amount of additional in-service education. Of the 14 sites proposed by the GoI that will offer the full 18 months post-nursing midwifery curriculum, only six are currently prepared to provide clinical experience in MLUs, with others anticipated to come on board shortly.

If local and federal investment in midwifery continues to be asymmetrical or parsimonious—in terms of funding and time allotted for acquisition of core competencies and professional development, the benefits sought in improved quality of care and maternal health outcomes through expansion of midwifery may not be realized. While the sense of urgency is appreciated, we strongly recommend that the roadmap for integration consider a renewed commitment to the educational standards that are well established and supported by global stakeholders.

Finally, according to both our desk review and KIs, developing a strong midwifery educator workforce is critical. Currently, international midwifery educators are filling that role, but growing a cohort of local senior midwifery educators will be key to culture congruency and credibility going forward. Caution should be used in having non-midwives, such as nurses and obstetricians, take on this educator role. Given the distinct knowledge, philosophy and approach midwives use to promote physiologic birth and respectful maternity care (RMC), this cannot be offered by other professional groups without risking discordant education.

### Gender and human rights considerations for midwifery services

In July 2019, a report was made to the United Nations recommending, among other things, that Nation States investigate and raise awareness about mistreatment and violence against women during reproductive health services and childbirth, in addition to establishing human rights based accountability mechanisms (United Nations General Assembly, 2019). In India, NPMs can play an integral part in screening and advocating for women and girls affected by gender-based inequities and violence, ensuring that all pregnant women are treated with respect. Conversely, childbearing people who are cared for by known midwives report increased ability to lead decisions about their care, more respectful care, less mistreatment, less pressure to accept interventions and fewer procedures without their consent compared with people under physician care (Vedam *et al.*, 2019a,b; Stoll *et al.*, 2021).

**Figure 4.**
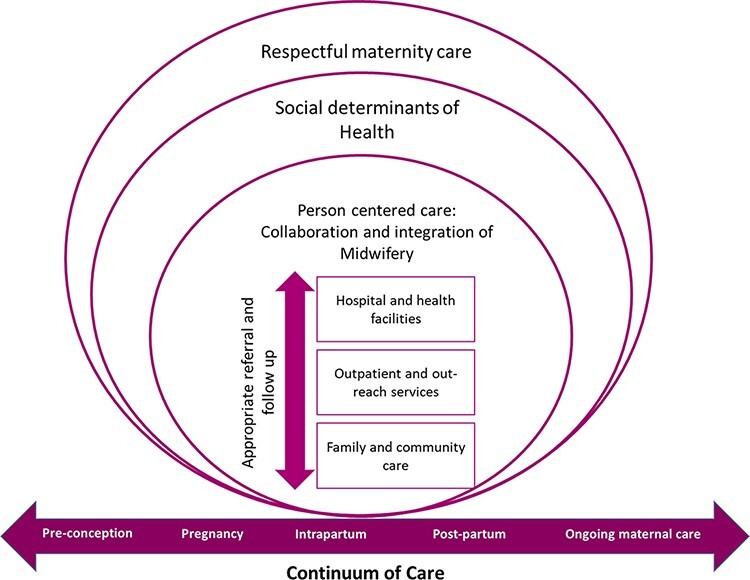
Midwifery continuum of care

Organizing health services in a way that centres the needs and perspectives of childbearing families could increase the utilization of services and skilled providers, such as midwives. Importantly, throughout India community-based maternity care and links to health facilities are primarily provided by ‘trusted’ front-line health workers, such as ASHAs. To align with an equity approach, given that marginalized communities likely have the greatest need but least access to the proposed MLUs, India could map the areas with poor NPM integration and place NPMs in regional health centres. NPMs could serve as a central resource to provide training and supplies to ASHAs, receive health information and/or provide primary care services or referrals to specialty care as needed. These NPMs could also provide family planning and safe abortion services, thus further expanding the healthcare systems’ ability to improve reproductive health outcomes and experiences (see Figure 4).

Finally, in practice human rights are invoked through both a set of legal frameworks—international, regional and local and specific accountability measures such as Human Rights Courts and Tribunals. National/local adoption, ratification and codification into regulatory and justice systems vary widely, affecting enforcement and accountability mechanisms. However, these frameworks, accountability measures and in-service training of all health professionals at point of service about the provision of trauma-informed care will be critical to service users and physician leaders’ understanding of the scope, role and potential of NPMs in India.

### Proactive public education

In the WWW campaign, we learned from Indian women across 27 states and territories that being treated with dignity and respect during their reproductive healthcare encounters was paramount. Unfortunately, this was often a response to their lived experiences of violations, disrespect, abuse and mistreatment during childbearing (Bohren *et al.*, 2015). Based on our synthesis we recommend a public education campaign that targets both service users (childbearing women) and healthcare providers (particularly obstetricians and nurses). Copious global evidence suggests that women in both high- and low-resourced countries perceive loss of autonomy and respect as lower quality of care. However, it is likely that if Indian women are used to poor care or mistreatment and an overly interventive medical approach to perinatal care and midwives offer a different model of care, they may not understand that respectful providers can also be skilled medical professionals.

Similarly, if there are multiple actors (such as nurses and obstetricians) defining midwifery scope of practice who are not familiar with the principles, philosophy, skills and practices of autonomous midwives, it will lead to greater tension and discordance between doctors, midwives and staff nurses. Given the existing hierarchy and infrastructure in the medical system, many KIs cited the challenge of shifting the perceptions of physicians. Proactive education of physicians about midwifery scope of practice will enhance their ability to recognize, welcome and appreciate midwives.

Securing acceptance of midwifery care requires intensive, clear and precise articulation of what midwives offer, who they are, how they are trained and how they can contribute to public health. Without this, midwifery scope of practice will be restricted or marginalized (Sharma *et al.*, 2013) and, consequently, risk attributing any lack of progress in population-level improvement in outcomes directly onto midwives.

### Enabling environments for midwifery integration

Our critical review underlines that healthcare facilities that build the pathway for midwives to practice to their full scope will be met with greater success (see Box 1). Potential pitfalls will appear if, after centralized training, NPMs return to facilities that have not been prepared or supported to welcome their return.

Clarifying the distinction between the unique expertise and roles of midwifery, nursing and obstetrics will be key to NPMs thriving in India. In one commonly used practice model each certified midwife works a 24-h in-hospital shift (Rosenstein *et al.*, 2015; Anil *et al.*, 2019). During their shift, each midwife provides care for an array of maternity patients. This may include provision of antenatal care; triaging women to admission or further medical evaluation; management of labour inductions, active labour patients or complications, following consultation with obstetricians and/or conducting postpartum rounds and facility discharges. Best practice models for midwifery show that midwives’ caseloads should be reduced if the case mix includes a higher proportion of clients with complex medical or social needs (e.g. from 36 to 26) (Hadebe *et al.*, 2021). Our review confirmed that in settings where midwives can address the full range of needs of childbearing women, independently managing cases and initiating referrals on their own recognizance, optimal outcomes ensue. On the other hand, if the individual midwife is expected to improve outcomes without concurrent priming of the healthcare system and facilities, conditions and outcomes for childbearing women will not improve (McFadden *et al.*, 2020).

Gender transformative policies that address the underlying causes of gender inequities and recognize the true value of women’s work, both paid and unpaid, will guarantee working conditions that support well-being, agency and human rights (WHO, 2016d, p. 55). The gender wage gap has been shown to be larger in health care than other sectors. It can be helpful to set wages of midwives in reference to the minimum wage for other health professionals, to identify gaps. For example, in Tunisia midwives are paid five times the minimum public sector wage, whereas general medical practitioners are paid 10 times the minimum wage (WHO, 2016d, p. 56).

Findings from both the desk review and interviews suggest that to help midwives cope with stress, high volume and critical incidents, they will need timely access to trauma-informed mental health support and protected time off. A work culture that normalizes seeking help and encourages self-care will be a safer and more sustainable work environment for midwives. There are points in a midwife’s life that make them more vulnerable to experience distress and leave the profession. These include early years of practice, having young children, experiencing a critical incident and ageing/developing chronic health issues (Sidhu *et al.*, 2020; Stoll and Butska, 2020). NPMs ideally ought to have the option of working in different models of care that suit their skill set and align with their personal circumstances and preferences. In addition, career diversification to various sectors (education, regulation, association, hospital leadership, health policy and quality assurance) could enable them to demonstrate leadership; enhance the profile of the profession and ensure that midwifery values and concerns permeate the workplace. Finally, a formalized re-entry strategy could minimize attrition of midwives following gaps in practice.

Our examination of the evidence also suggests that, to avoid some common pitfalls in retention suffered by other countries, India should build the necessary professional structures and pathways to midwifery leadership roles. A clear career progression plan can contribute to the professional identity formation of the NPM cadre and to recruitment of high-calibre candidates. Senior NPMs and midwife leaders can, in turn, provide role modelling for junior colleagues. These clinical mentors will be key in helping the next generation of NPMs to envision their role as skilled, autonomous professionals. KIs stressed that midwives and physicians provide different services to the health system, and one profession cannot train the other. Simply ‘following doctor’s orders’ will not build the critical and independent thinking and practice needed to run midwifery-led units. If midwives practice alone without mentoring or professional community, it will be difficult to implement the midwifery care model.

A collaborative model between NPMs and consultant can be operationalized in the proposed MLUs through regular multidisciplinary meetings where reviews of cases are led alternately by different team members within a peer learning and supportive framework; evidence that supports each profession’s approach is shared and differences are acknowledged in a transparent manner. Midwife-led training of obstetric residents and nurses and midwifery trainee clinical placements in acute care settings can contribute to mutual professional trust and appreciation (Angelini *et al.*, 2012). In places that are utilizing these strategies, there are notable decreases in operative delivery rates, better neonatal outcomes (Anil *et al.*, 2019; Kenyon *et al.*, 2016) and greater patient satisfaction.

### Evaluating success

When midwives are newly regulated and integrated into regional healthcare systems, significant inter-professional conflict can persist around recommendations for safe birth care unless data can establish improved outcomes. Evidence of the benefits that midwives bring to maternal and neonatal outcomes may serve both short-term and long-term goals to help midwives gain the recognition and respect they deserve.

The Access and Integration Maternity care Mapping study examined associations among regulation, scope of practice and inter-professional collaboration with maternal–newborn outcomes and equitable care for at-risk populations in the USA (Vedam *et al.*, 2018). A team of transdisciplinary experts detailed differences across jurisdictions in scope of practice, autonomy, governance and coordination of care that affect access to safety and quality. The investigators calculated correlations between midwifery integration scores (MISS) and selected outcomes (e.g. spontaneous vaginal birth, vaginal birth after cesarean, breastfeeding, Caesarean, induction, neonatal mortality and low birth weight) in each state, controlling for the type of provider. State MISS scores demonstrated the positive impact of effective models for integration on population-level maternal–newborn health.

Similarly, by evaluating the effects of access to midwives and the lived experience of childbearing women before and after implementation of midwives, the GoI can identify gaps in gender equity and in maternal–newborn outcomes and quality of care. If the national health system commits to uncompromising standards for RMC, they will also implement the routine use of person-centred indicators (Vedam *et al.*, 2017a,b; 2019b; Bohren *et al.*, 2018) to ensure that all cadres are prioritizing autonomy, respect and unconditional positive regard for service users.

## Limitations

Desk reviews are by nature an exploratory method and often does not have an explicitly defined search strategy; hence, there is the possibility of missing some relevant literature around the topic. For example, our search strategy may not have covered the diverse terminology used to refer to the midwife and midwifery model of care. We have attempted to be transparent about our strategy and inclusion/exclusion criteria with the addition of the Supplementary Table (S1). While our desk review may have excluded some key documents, the rapid saturation of our interview findings suggests that we were unlikely to have missed a key theme in our desk review results. Although written by a group of experts with a detailed and well-grounded knowledge of the issues, the results may also be influenced by the reviewer’s theories, needs and beliefs.

In addition, a referral and networking method for the identification of KIs may have inadvertently excluded experts who have different perspectives. While we deliberately identified and interviewed informants from different regions and professional roles, since many of the factors described (resource problems and hierarchy) are subject to local context, the KIs may not identify the full range of existing structural barriers. We do not claim that the issues raised in the interviews are generalizable to all settings—as qualitatively oriented data collection is focused on gathering the view of specific people with expertise and insight on the phenomena of interest. Similarly, since we intended to explore varied dimensions of the creation of a new midwifery cadre, which has not yet been widely accepted throughout India, this review may not have captured issues related to implementation and acceptability that will be encountered. Finally, implementation of midwifery education and workforce models is rapidly changing in India—some recommendations may already have been actioned.

## Conclusion

In summary, prioritizing a full-scope relationship-based model of care for the new cadre of NPMs in India will specifically address the health needs of women and girls including equitable, rights-based approaches to care during and after pregnancy and birth. Midwifery leadership is essential to building a strong, resilient, independent and respectful workforce. The proposed programmes for MLUs can be developed with a clear understanding and recognition of gender transformative work policies that create good working conditions for midwives and close gender gaps in pay and leadership. Any attempt to ‘scale up’ the NPM training programmes requires that the facilities where they work will be prepared for this practice model—issues discussed above such as enabling working conditions, educating other clinicians, public messaging for clients, ongoing mentoring and training in evidence-based care—will all feed the success of a midwifery-led care in India. In addition, without enabling environments, midwives will not be positioned to create the changes needed to make RMC the standard of care throughout India. We present ways to organize midwifery work that optimize well-being and sustainable practice and can reduce gender inequity in the workplace.

A commitment to unconditional RMC and freedom from abuse from front-line community health workers, facility intake personnel, nurse, midwives and physicians is essential to ensure safety for all families and will contribute to increased trust, uptake of services and optimal maternal–newborn outcomes (Miller *et al.*, 2016; Bohren *et al.*, 2020). Most importantly at the regional, state, community and facility levels health human resource planners should ensure that local women are involved at all levels of decision-making, development, implementation and evaluation of the proposed midwifery programme.

## Supplementary Material

czac032_Supp

## Data Availability

The data underlying this article are available in the article and in its online supplementary material.
