# Supplementary material for: Advancing quality and safety of perinatal services in India: opportunities for effective midwifery integration
Source: Health Policy Plan. 2022 Apr 16;37(8):1042–63. doi: 10.1093/heapol/czac032 (PMC9469892; doi:10.1093/heapol/czac032)
Supplement: czac032_Supp [file czac032_supp.zip › Supplementary Table S1_Final BW.doc]

**Table S1:** Summary of included studies

| **Authors** | **Year** | **Country** | **Nature of document** | **Purpose/ Aim** |
| --- | --- | --- | --- | --- |
| World Health Organization [43] | 2016 | Geneva, Switzerland | Policy document | Recommendations on antenatal care for a positive pregnancy experience |
| UNHCHR, Harvard  FXB Center for Health and Human Rights, the Partnership for MNCH, UNPF and WHO [44] | 2016 | - | Report | Application of human rights-based approach to sexual and reproductive health, maternal health and under-5 child health |
| Miller S, Abalos E, Chamillard M et al. [11]. | 2016 | Global | Peer-reviewed publication | Describes the two extreme situations in relation to respectful maternity care worldwide |
| Chou VB, Walker N,  Kanyangarara M [12] | 2019 | LMICs | Research article | Estimate the global impact of poor quality of care on maternal and neonatal outcomes in 81 low- and middle-income countries |
| World Health Organization [45] | 2016 | Geneva, Switzerland | Working paper | Normative guidance document developed to support maternal, newborn and child quality of care improvement |
| The Partnership for Maternal Newborn & Child Health [46] | 2017 | - | Press release | Launch of Network for Improving Quality of Care: Professional associations affirm commitment to improving the quality of maternal and newborn health care. |
| Shrivastava S, Sivakami M [20] | 2020 | India | Integrative review | Review of the existing literature in India on violence against women during childbirth |
| Ginsburg FD, Rapp R [48] | 1995 |  | Book | Explores the complexity of contemporary sexual politics around the globe |
| Jordan B [49] | 1992 | - | Book | Identify factors providing safe outcomes for the mother and infant |
| Oakley A. [50] | 1980 | United Kingdom |  | Sociology of childbirth |
| Carter M [51] | 2012 | US | Review | Overview of health center regulations and policies affecting midwives |
| Center for Health workforce studies [52] | 2015 | New York | Report | Primary care workforce capacity and selected characteristics. |
| Raisler J, Kennedy HP [53] | 2005 | US | Systematic literature review | review of research on midwifery care of poor and vulnerable women |
| Sword W, Heaman MI, Brooks S et al. [54] | 2012 | Canada | Qualitative research article | Explore women's and care providers' perspectives of quality prenatal care |
| Vedam S, Stoll K, Martin K et al. [55] | 2017 | BC, Canada | Research article | Develop and validate a new instrument that assesses women's autonomy and role in decision making during maternity care |
| Kennedy HP, Rousseau AL, Low LK. [56] | 2003 | US | Research article | Provide valuable insight and knowledge about the distinctive aspects of midwifery care and how they may be related to specific outcomes. |
| Sword W [57] | 2003 | Canada | Research article | Explanation of prenatal care use among women of low income |
| Downe S, Finlayson K, Tunçalp et al. [58] | 2016 | Global | Scoping review | Identify the processes and outcomes of antenatal care provision that are important to healthy pregnant women |
| Homer CSE, Friberg IK, Dias MAB et al. [25] | 2014 | Global | Peer reviewed publication | Examine the projected effect of scaling up  midwifery |
| Hunter B, Berg M, Lundgren I et al.[59] | 2008 |  | Commentary | Explore importance of relationship of maternity care provider and mothers |
| Hodnett ED, Gates S, Hofmeyr GJ et al. [60] | 2011 | HIC and MIC | Systematic review | Assess the effects on women and their babies, of continuous, one-to-one intrapartum support compared with usual care |
| Kennedy HP, Balaam M-C, Dahlen H et al. [30] | 2020 | United states | Peer-reviewed publication | The role of midwifery and other  international insights for maternity care in the United States |
| Ten Hoope-Bender P, De Bernis L, Campbell J, Downe S, Fauveau V, Fogstad H, et al [27] | 2014 |  | Peer-reviewed publication | The potential effect of life-saving interventions and the sequence of health system changes that made a reduction in maternal mortality possible in countries that have expanded their midwifery workforce |
| Leonard TMB [61] | 2017 |  | Book chapter | Expanding the social justice discourse surrounding reproductive rights |
| Ross L [62] | 2017 |  | Book chapter | expansion on social justice discourse explores practical applications for activist thought migrating from the community |
| Renfrew MJ, McFadden A, Bastos MH, Campbell J, Channon AA, Cheung NF, et al. [24] | 2014 | Global | Peer-reviewed publication | Map the scope of midwifery and develop a framework for quality maternal and newborn care including synthesis of findings from systematic reviews of women's views and experiences, effective practices, and maternal and newborn care providers |
| McRae DN, Janssen PA, Vedam S, Mayhew M, Mpofu D, Teucher U, et al. [63] | 2018 | Canada | Peer-reviewed publication | Investigate if antenatal midwifery care was associated with lower odds of small-for-gestational-age birth, preterm birth or low birth weight compared with general practitioner or obstetrician models of care |
| Sandall J, Soltani H, Gates S, Shennan A, Devane D. [66] | 2015 |  | Research article | Compare midwife-led continuity models of care with other models of care for childbearing women and their infants. |
| Walsh D, Spiby H, Grigg CP *et al.* [67] | 2018 | United Kingdom | Peer-reviewed publication | Describe the types, numbers, and utilization of midwifery (alongside and free-standing) and obstetric units in England. |
| Stapleton SR, Osborne C, Illuzzi J. [68] | 2013 | United States | Peer-reviewed publication | Prospective cohort study examining the safety of midwifery-led birth center model of collaborative care. |
| Christensen LF, Overgaard C. [69] | 2017 | Denmark | Peer-reviewed publication | Matched cohort study comparing outcomes between freestanding midwifery units and obstetric units. |
| Baczek G, Tataj-Puzyna U, Sys D *et al.* [70] | 2020 | Global | Peer-reviewed publication | Narrative review of freestanding midwife-led units, including outcomes and the structure of MLUs. |
| Long Q, Allanson ER, Pontre J *et al.* [71] | 2016 | Global | Peer-reviewed publication | Systematic review of the impacts of onsite midwife-led birth units (OMBUs) embedded within hospitals, compared to standard obstetric care. In low- and middle-income settings, OMBUs may be a safe and cost-effective model. |
| Macdonald D, Snelgrove-Clarke E, Campbell-Yeo M *et al.* [77] | 2015 | Global | Peer-reviewed publication | Systematic review of qualitative evidence around the experiences of midwives and nurses collaborating to provide birthing care. Identified challenge areas for collaboration and implications for practice. |
| Raab CA, Will SEB, Richards SL *et al.* [78] | 2013 | United States | Peer-reviewed publication | Overview of a collaborative obstetric patient safety initiative implemented in three academic facilities. Affirms importance of Interprofessional collaboration in achievement of positive health outcomes. |
| Liberati EG, Tarrant C, Willars J *et al.* [79] | 2019 | United Kingdom | Peer-reviewed publication | Ethnographic study of high-performing maternity unit to explore mechanisms and factors that contribute to safe outcomes. |
| Peterson WE, Medves JM, Davies BL *et al.* [80] | 2007 | Canada | Peer-reviewed publication | Attitudes and beliefs of care providers towards delivery of multidisciplinary collaborative maternity care. Strategies to encourage collaboration are discussed. |
| Bartlett L, Weissman E, Gubin R *et al.* [81] | 2014 | Global | Peer-reviewed publication | Simulation estimating lives and costs if midwifery and obstetrics services are scaled up in 58 low- and middle-income countries. |
| Vedam S, Stoll K, MacDorman M *et al.* [82] | 2018 | United States | Peer-reviewed publication | Measures association between midwifery integration (using MISS scoring system) and pregnancy and birth outcomes. The regulatory environment has a meaningful impact on patient access and quality of care. |
| Behruzi R, Klam S, Dehertog M *et al.* [83] | 2017 | Canada | Peer-reviewed publication | Identifies interactional, organizational, and systems-level factors that influence interprofessional collaboration between midwives and other health care providers. |
| Waldman R, Kennedy HP, Kendig S. [84] | 2012 | United States | Book chapter | Issues and practical suggestions to the maternity workforce crisis are discussed with particular focus on collaboration among different types of care providers. |
| Marshall JL, Spiby H, McCormick F. [85] | 2015 | United Kingdom | Peer-reviewed publication | Evaluation of a program intended to promote physiologic birth and reduce unnecessary caesarean section rates. |
| World Health Organization [74] | 2016 | Global | Report | Presentation of findings based on global consultation of midwifery personnel; reflections on critical issues and barriers experienced in care provision. |
| Kristensen TS, Borritz M, Villadsen E *et al.* [87] | 2005 | Denmark | Peer-reviewed publication | Introduction of the Copenhagen Burnout assessment tool and presentation of burnout scores among professions in human service sector. |
| Hall LH, Johnson J, Watt I *et al.* [88] | 2016 | Global | Peer-reviewed publication | Systematic review presenting association between moderate to high burnout among healthcare professionals’ and patient safety outcomes. |
| Shanafelt TD, Noseworthy JH [89] | 2017 | United States | Commentary | Nine organizational strategies are proposed to promote physician engagement and reduce burnout; reflections on implementation experience are discussed. |
| Stoll K, Gallagher J. [90] | 2019 | Canada | Peer-reviewed publication | Survey of burnout among midwives in Western Canada. |
| Royal College of Midwives [91] | 2016 | United Kingdom | Report | Updated report on factors that result in career departure of midwives, including workload, staffing shortages, and dissatisfaction with pay scale. |
| Stoll K, Butska L, Sustainable Midwifery Practice Taskforce [92] | 2020 | Canada | Report | Report of findings from the Sustainable Midwifery Task Force on solutions to occupational stress and burnout among midwives practicing in British Columbia, Canada. |
| Cramer E, Hunter B. [93] | 2018 | Global | Review paper | Review of risks to emotional well-being of midwives and identification of modifiable factors and priorities. |
| Sidhu R, Su B, Shapiro KR *et al.* [94] | 2020 | Global | Scoping review | Scoping review of burnout within the midwifery profession, with attention to the prevalence, contributing factors, and potential solutions. |
| Cull J, Hunter B, Henley J *et al.* [95] | 2020 | United Kingdom | Peer-reviewed publication | Qualitative analysis of factors contributing to career satisfaction and dissatisfaction among early-career midwives. |
